# Hepatic Angiomyolipoma With Predominant Lipomatous Component: A Rare Entity

**DOI:** 10.7759/cureus.54357

**Published:** 2024-02-17

**Authors:** Jaweria Pervaiz, Samina Zaman, Sohaib Khalid, Zubaria Rafique, Rida Noor

**Affiliations:** 1 Department of Histopathology, Chughtai Institute of Pathology, Lahore, PAK; 2 Department of Histopathology, Children's Hospital and Institute of Child Health, Lahore, PAK; 3 Department of Pathology, Faisalabad Medical University, Faisalabad, PAK

**Keywords:** angiomyolipoma, hepatic, lipomatous predominant, pecomas, rare entity

## Abstract

Hepatic angiomyolipoma (HAML) is a rare benign mesenchymal tumor with varying amounts of mature adipose tissue, smooth muscle cells, and thick-walled blood vessels. We present a rare case of hepatic angiomyolipoma (AML) with predominant lipomatous components. A 42-year-old female presented to the hospital with pain in the right lumbar region. On imaging, there was a large fat-predominant mass attached to the surface of the liver extending down to the lumbar region. On small biopsy, it was reported as a well-differentiated adipocytic neoplasm, and fluorescence in situ hybridization (FISH) studies performed for *MDM2* were negative. On excision, histopathological examination showed predominantly fat components, but there were few epithelioid cells between adipocytes and thick-walled blood vessels. These cells were positive for Melan-A, HMB45, and smooth muscle actin (SMA) and negative for hepatocyte paraffin-1 (Hep Par1). Angiomyolipoma is a benign tumor and has a good prognosis with surgical excision. Few cases are associated with tuberous sclerosis.

## Introduction

Hepatic angiomyolipoma (HAML) is a rare benign mesenchymal tumor with varying amounts of mature adipose tissue, smooth muscle cells, and thick-walled blood vessels. HAML belongs to the perivascular epithelioid cell tumor (PEComa) family of tumors. The perivascular epithelioid cell family of tumors (PEComas), defined by their co-expression of melanocytic and muscle markers, includes angiomyolipoma (AML), lymphangioleiomyoma (LAM), and clear cell "sugar" tumors of the lung, pancreas, and uterus first proposed by Bonetti et al. in 2003 [1]. Other than angiomyolipoma (AML) and lymphangioleiomyoma (LAM), the majority of PEComas are sporadic, and only a small number of PEComas are directly associated with the genetic alterations of tuberous sclerosis. PEComas may have developed as *TSC2*-associated neoplasm due to the tuberous sclerosis complex (TSC) and deletion of 16p, the location of the *TSC2* gene [2].

## Case presentation

A 42-year-old female patient presented to the hospital with pain in the right lumbar region. On physical examination, a palpable mass was found in the upper right quadrant of her abdomen. Her clinical history showed no signs of tuberous sclerosis. Her blood counts were normal. Liver function tests showed a slight increase in serum levels of aspartate transaminase, alanine transaminase, and gamma-glutamyl transferase. Moreover, serum viral hepatitis markers, including hepatitis B antigen and anti-hepatitis C virus antibody, were negative. Serologies for alpha-fetoproteins, serum CA19-9 level, serum beta-human chorionic gonadotropin (HCG) level, and serum *Echinococcus* IgG were normal.

**Figure 1:**
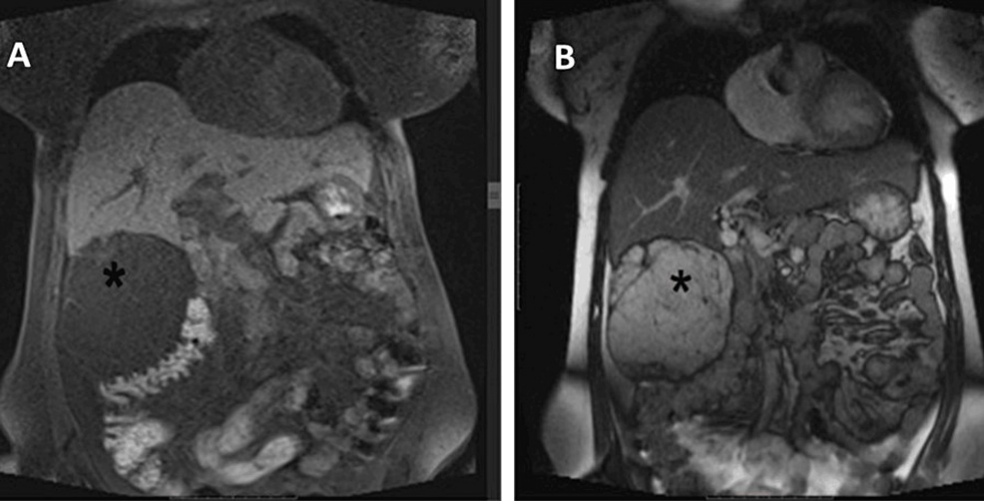
(A) Coronal T1 fat-suppressed and (B) coronal T2WI demonstrate the liver as the organ of origin with mass (marked with an asterisk) showing suppression of signals on fat, thin internal septae and soft tissue. T2WI: T2-weighted image

**Figure 2:**
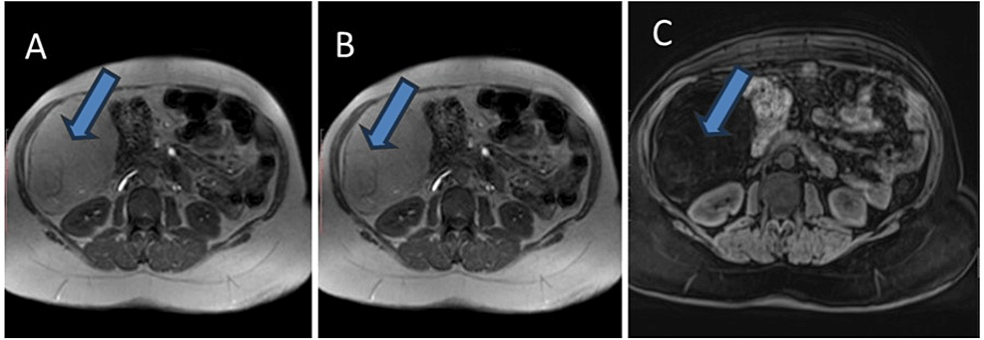
(A, B, and C) T2, T1, and T1 fat-suppressed sequences show large hyperintense lesion (blue arrow) suppression of signals.

Contrast-enhanced computed tomography (CECT) of the abdomen and pelvis (Figures 1, 2) revealed a well-circumscribed predominantly fatty mass in the abdomen measuring 107×104×101 mm, extending from the level of the inferior surface of the liver superiorly to the right lumbar region inferiorly. The mass was inferomedially displacing the hepatic flexure of the colon and transverse colon along with indentation of the inferior surface of the right lobe of the liver. The initial observation revealed the presence of a fatty mass located in the right hemi-abdomen, with a differential diagnosis primarily considering angiomyolipoma; consequently, biopsy correlation was recommended.

Upon microscopic examination of the small biopsy specimen, it was documented as a well-differentiated adipocytic neoplasm. Fluorescence in situ hybridization (FISH) studies for *MDM2* gene amplification were negative, ruling out liposarcoma. However, for a definitive diagnosis, a partial hepatectomy was done. The patient remained in the hospital for four days postoperatively. Now, the patient is in the usual state of health.

**Figure 3:**
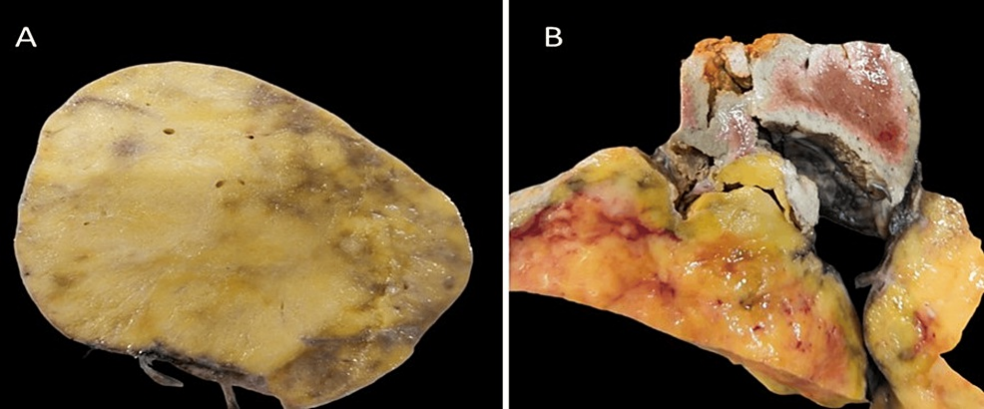
(A) Unencapsulated tumor with yellowish firm cut surface and (B) demarcation with normal liver parenchyma visible.

A gross examination of hepatectomy (Figure 3) showed an unencapsulated tumor, clearly demarcated from the surrounding non-cirrhotic liver. All surgical margins were free of tumors.

**Figure 4:**
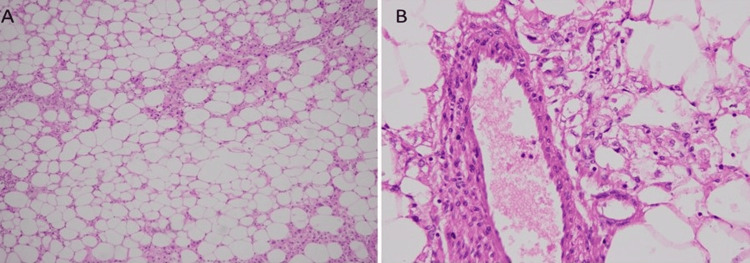
(A) Tumor shows predominantly adipocytic component (B) on high power admixed with epithelioid cells arranged around thick-walled blood vessels.

Histopathological analysis disclosed a predominantly lipomatous component, interspersed with occasional epithelioid cells, blood vessels, and spindled cells, as illustrated in Figure 4.

**Figure 5:**
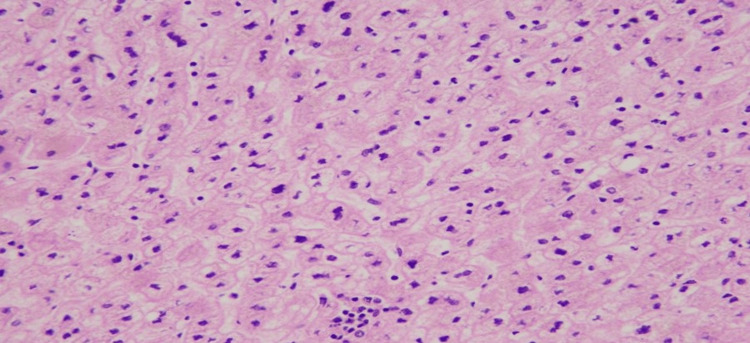
Tumor with areas of extramedullary hematopoiesis.

Areas of extramedullary hematopoiesis were also noted as depicted in Figure 5.

**Figure 6:**
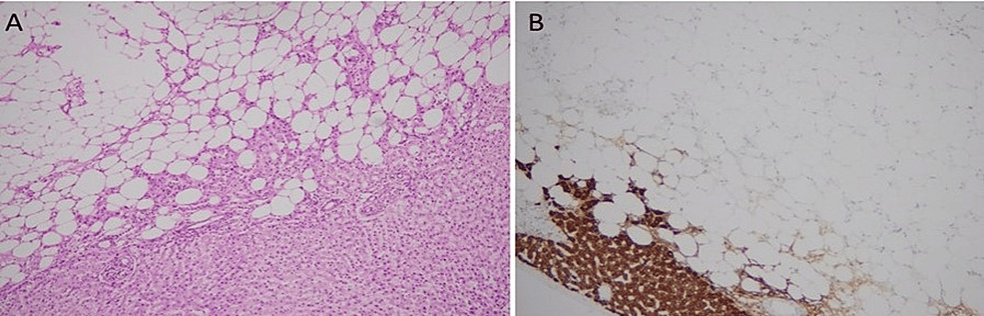
(A) Microscopic image of hepatic angiomyolipoma, fat predominant, with normal liver and (B) Hep Par1 immunostain positive in normal liver and negative in tumor cells.

**Figure 7:**
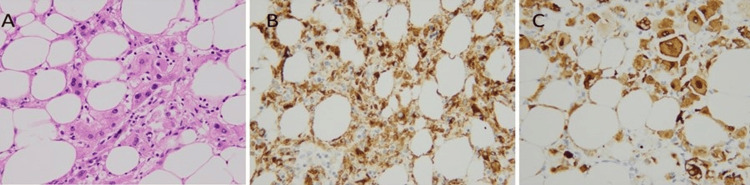
(A) Epithelioid smooth muscle cells (B) positive for HMB45 and (C) Melan-A immunostains.

**Figure 8:**
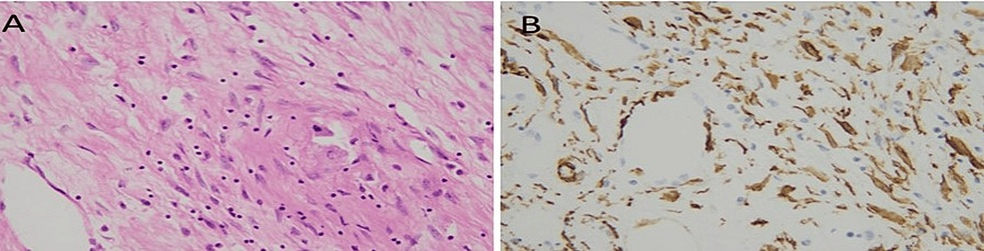
(A) Spindled smooth muscle component (B) highlighted by SMA stain. SMA: smooth muscle actin

Tumor cells were positive for melanocytic (Melan-A and HMB45) and smooth muscle actin (SMA) markers and negative for hepatic (hepatocyte paraffin-1 (Hep Par1)) marker as shown in Figures 6-8. Keeping in view the clinical history, gross and microscopic features, and results of immunostains, a final diagnosis of hepatic angiomyolipoma with predominant lipomatous component was rendered.

## Discussion

HAML comprises blood vessels, smooth muscle cells, and fat, but any component can predominate, leading to a wide differential diagnosis. According to the number of predominant components, AML is categorized as mixed (the most common type), lipomatous (with more than 70% fat component), myomatous (10% fat), and angiomatous.

Previously, a HAML case has been reported with a trace amount of fat, with a radiological differential of hepatocellular carcinoma [3]. Our case showed a lesion with fat predominance with a differential of angiomyolipoma on radiology and small biopsy reported as adipocytic neoplasm. Our case study is different because fat predominance in a tumor raised a differential of adipocytic neoplasm on gross examination and low-power microscopic examination.

Most renal angiomyolipomas present with complications of rupture and hemorrhage, especially those with size >4 cm or intralesional aneurysm >5 mm [4]. However, hepatic AML is mostly asymptomatic with vague symptoms such as abdominal dullness and pain, and rarely with rupture and hemorrhage, mostly among middle-aged females [5]. Only 600 cases of hepatic AML are reported in the literature.

According to a comprehensive review, many hepatic AMLs are benign and do not require resection [6]. Correct diagnosis of this rare benign lesion is important because misdiagnosis can lead to drastic treatment-related consequences. A multi-institutional study showed that a diagnostic biopsy should be performed to rule out hepatic AML when cross-sectional radiology shows a lesion with intralesional fat and washout in a non-cirrhotic liver. If the biopsy shows an uncertain diagnosis or malignant pattern, then excision or partial hepatectomy is recommended [7].

In the case of biopsy-confirmed HAML, conservative management with regular imaging follow-up is recommended, because there is no risk of malignant transformation [7]. As in our case, on a small biopsy, it was reported as adipocytic neoplasm, so resection was done after FISH studies for *MDM2* gene mutation. FISH studies negative for *MDM2* gene mutation rule out liposarcoma. It has been reported that 4% of patients with HAML exhibit malignant behavior, including (invasive) growth, recurrence after surgical resection, and even metastasis. These features were not seen in our case study patient. Most epithelioid-type HAMLs are thought to exhibit this malignant behavior but without any distinct molecular alteration [6].

## Conclusions

To summarize, hepatic angiomyolipoma (HAML) is a rare benign liver tumor composed of varying portions of smooth muscle cells, adipose tissue, and blood vessels. In our case, we had a patient with a predominant lipomatous component, which is an unusual entity, and the course of management and treatment remained the same. The patient underwent partial hepatectomy and has no postoperative complications. The presence of smooth muscle cells is particularly distinctive for its diagnosis and typically exhibits positive staining with HMB45 and Melan-A. HAML is a positive myomelanocytic marker, which helps in differentiating it from other liver lesions. Treatment for hepatic AML typically involves complete surgical removal, and it does not display a tendency for metastasis or malignant transformation.
